# EVIDENCE-DRIVEN PROGRAMS IN THE HEALTHCARE SETTING: HOW CAREGIVERS CAN BENEFIT

**DOI:** 10.1093/geroni/igz038.1651

**Published:** 2019-11-08

**Authors:** Christine J Jensen, Christine J Jensen

**Affiliations:** Riverside Center for Excellence in Aging and Lifelong Health, Williamsburg, Virginia, United States

## Abstract

How do family caregivers who interface with the healthcare system benefit from evidence-driven programs designed to support them? Education, training, and care coordination programs have all been found to significantly improve the well- being of caregivers. These programs include Operation Family Caregiver; Caring For You, Caring For Me; BRI Care Consultation; the New York University Caregiver Intervention; and patient and family advisory councils. Healthcare systems that host, translate and scale these programs make them more readily accessible for patients and their families, staff, and the larger community. Further, there is legislation, including the CARE Act, which recognizes the key role a family caregiver provides as a partner in their love one’s care. The CARE Act, now enacted in 36 states, states that family caregivers must be identified in the medical record and provided with the necessary resources to be able to care for their loved one after a hospital stay.

